# Multiresponsive 4D Printable Hydrogels with Anti-Inflammatory Properties

**DOI:** 10.1021/acsmacrolett.4c00404

**Published:** 2024-08-14

**Authors:** Maria Regato-Herbella, Daniele Mantione, Agustín Blachman, Antonela Gallastegui, Graciela C. Calabrese, Sergio E. Moya, David Mecerreyes, Miryam Criado-Gonzalez

**Affiliations:** † POLYMAT University of the Basque Country UPV/EHU, Joxe Mari Korta Center. Avda. Tolosa 72, 20018, Donostia-San Sebastián, Spain; ‡ Center for Cooperative Research in Biomaterials (CIC biomaGUNE), Basque Research and Technology Alliance (BRTA). Paseo de Miramón 194, 20014, Donostia-San Sebastián, Spain; § Ikerbasque, Basque Foundation for Science, 48013 Bilbao, Spain; ∥ Universidad de Buenos Aires, Facultad de Farmacia y Bioquímica, Departamento de Ciencias Biológicas, Junín 956, 1113 Ciudad Autónoma de Buenos Aires, Buenos Aires C1053ABH, Argentina

## Abstract

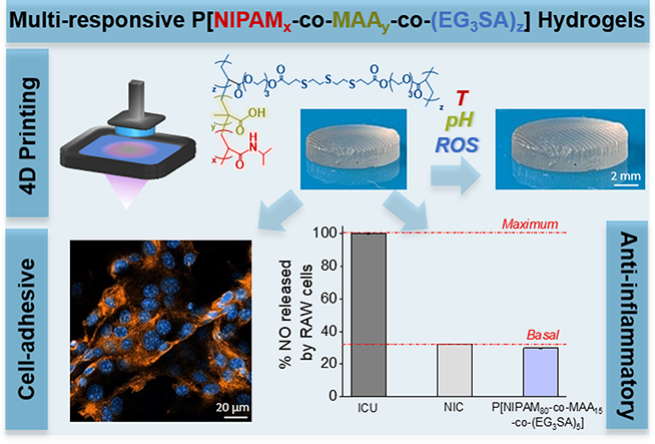

Multiresponsive hydrogels are valuable as biomaterials due to their ability to respond to multiple biologically relevant stimuli, i.e., temperature, pH, or reactive oxygen species (ROS), which can be present simultaneously in the body. In this work, we synthesize triple-responsive hydrogels through UV light photopolymerization of selected monomer compositions that encompass thermoresponsive *N*-isopropylacrylamide (NIPAM), pH-responsive methacrylic acid (MAA), and a tailor-made ROS-responsive diacrylate thioether monomer (EG_3_SA). As a result, smart P[NIPAM_*x*_-*co*-MAA_*y*_-*co*-(EG_3_SA)_*z*_] hydrogels capable of being manufactured by digital light processing (DLP) 4D printing are obtained. The thermo-, pH-, and ROS-response of the hydrogels are studied by swelling tests and rheological measurements at different temperatures (25 and 37 °C), pHs (3, 5, 7.4, and 11), and in the absence or presence of ROS (H_2_O_2_). The hydrogels are employed as matrixes for the encapsulation of ketoprofen (KET), an anti-inflammatory drug that shows a tunable release, depending on the hydrogel composition and stimuli applied. The cytotoxicity properties of the hydrogels are tested *in vitro* with mouse embryonic fibroblasts (NIH 3T3) and RAW 264.7 murine macrophage (RAW) cells. Finally, the anti-inflammatory properties are assessed, and the results exhibit a ≈70% nitric oxide reduction up to base values of pro-inflammatory RAW cells, which highlights the anti-inflammatory capacity of P[NIPAM_80_-*co*-MAA_15_-*co*-(EG_3_SA)_5_] hydrogels, *per se*, without being necessary to encapsulate an anti-inflammatory drug within their network. It opens the route for the fabrication of customizable 4D printable scaffolds for the effective treatment of inflammatory pathologies.

Multiresponsive hydrogels, also known as intelligent or smart hydrogels, can undergo controlled shape changes in response to more than one stimulus, which has attracted great attention in the biomedical field for drug delivery,^1^ tissue engineering,^2,3^ cancer therapy,^4^ or biosensing.^5,6^ Their ability to change their properties upon response to biological (i.e., temperature, pH, enzyme activity, reactive oxygen species), and/or external stimuli, (i.e., light, electrical or magnetic field),^7−9^ make them ideal candidates for 4D printing, a cutting-edge technology for manufacturing customizable dynamic materials combining 3D printing and stimuli-responsiveness.^10,11^

The design of tailor-made stimuli-responsive polymers allows to provide specific and simultaneous responses to different stimuli, a common scenario in biology.^12−16^ Poly(*N*-isopropylacrylamide) (PNIPAM) is the most studied polymer to develop thermoresponsive hydrogels. PNIPAM displays a reversible volume phase transition through swelling at temperatures below the so-called lower critical solution temperature (LCST ∼ 32 °C) and shrinking above it.^3^ This swelling/shrinking process has been modulated through copolymerization with other monomers, which in turn confer responsiveness to other environmental stimuli such as pH.^12^ For example, copolymers of PNIPAM and methacrylic acid (MAA) or acrylic acid (AA) can be deprotonated at high pHs, above their p*K*_a_, endowing hydrogels with pH-response in addition to temperature sensitivity.^17^ PMAA and PAA hydrogels have also been exploited for targeted drug release as they acted as drug protectors at acidic conditions in the stomach to be later released at higher pH ∼ 8 in the gastrointestinal tract.^18^ The dual response of P[NIPAM-*co*-MAA] and P[NIPAM-*co*-PAA] copolymers to temperature and pH changes has also been studied by many authors for controlled drug release.^19−21^ More recently, the use of reactive oxygen species (ROS), oxidant species present in the human body, has drawn attention as possible stimuli for responsive hydrogels. ROS effect can vary from beneficial cell survival to nondesirable oxidative stress when they are overproduced, thus causing inflammation, cancer, and age-related diseases.^22,23^ Among different types of ROS-responsive polymers (i.e., sulfides, diselenides, thioketals, aryl boronic esters, etc.), those bearing thioether groups present interesting hydrophobic to hydrophilic transitions when oxidized by ROS without requiring cleavage.^24−27^ Very recently, we developed ROS-responsive photopolymerizable thioether-based hydrogels through the synthesis of aqueous soluble redox monomers from oligomers of ethylene glycol sulfur diacrylate (EG_3_SA). The resulting hydrogels were used as 5-Fluorouracil carriers to inhibit the growth of melanoma cancer cells.^28^

Considering that overproduction of ROS in tumor/inflamed areas is generally linked to pH changes becoming slightly acidic (pH 5.4–7.1),^29,30^ the development of intelligent hydrogels that respond simultaneously to ROS, acidic pH, and body temperature is an interesting approach to modulate the simultaneous multistimulation in complex biological environments. Here, we have synthesized multiresponsive hydrogels through UV-light photopolymerization of a selected monomers mixture consisting of thermoresponsive NIPAM, pH-responsive MAA, and a tailor-made ROS-responsive EG_3_SA monomer. P[NIPAM_*x*_-*co*-MAA_*y*_-*co*-(EG_3_SA)_*z*_] hydrogels’ response to external stimuli (temperature, pH, and ROS) and controlled-release properties are investigated. Their additive manufacturing through digital light processing (DLP) 4D printing is also performed to obtain customizable hydrogels. Finally, we show that the hydrogels display anti-inflammatory properties.

P[NIPAM_*x*_-*co*-MAA_*y*_-*co*-(EG_3_SA)_*z*_] hydrogels were synthesized by copolymerization of three different responsive monomers, thermoresponsive NIPAM (*x* = 80, 70 or 40%mol), pH-responsive MAA (*y* = 15, 15 or 20%mol), and ROS-responsive EG_3_SA (*z* = 5, 15 or 40%mol). Then, the hydrogels were obtained by UV-light photopolymerization (λ = 365 nm) using Darocur 1173 (2-hydroxy-2-methylpropiophenone) as a photoinitiator and 30 wt % CHCl_3_ as a sacrificial solvent (Figure 1). In all cases, transparent hydrogels were formed.

**Figure 1:**
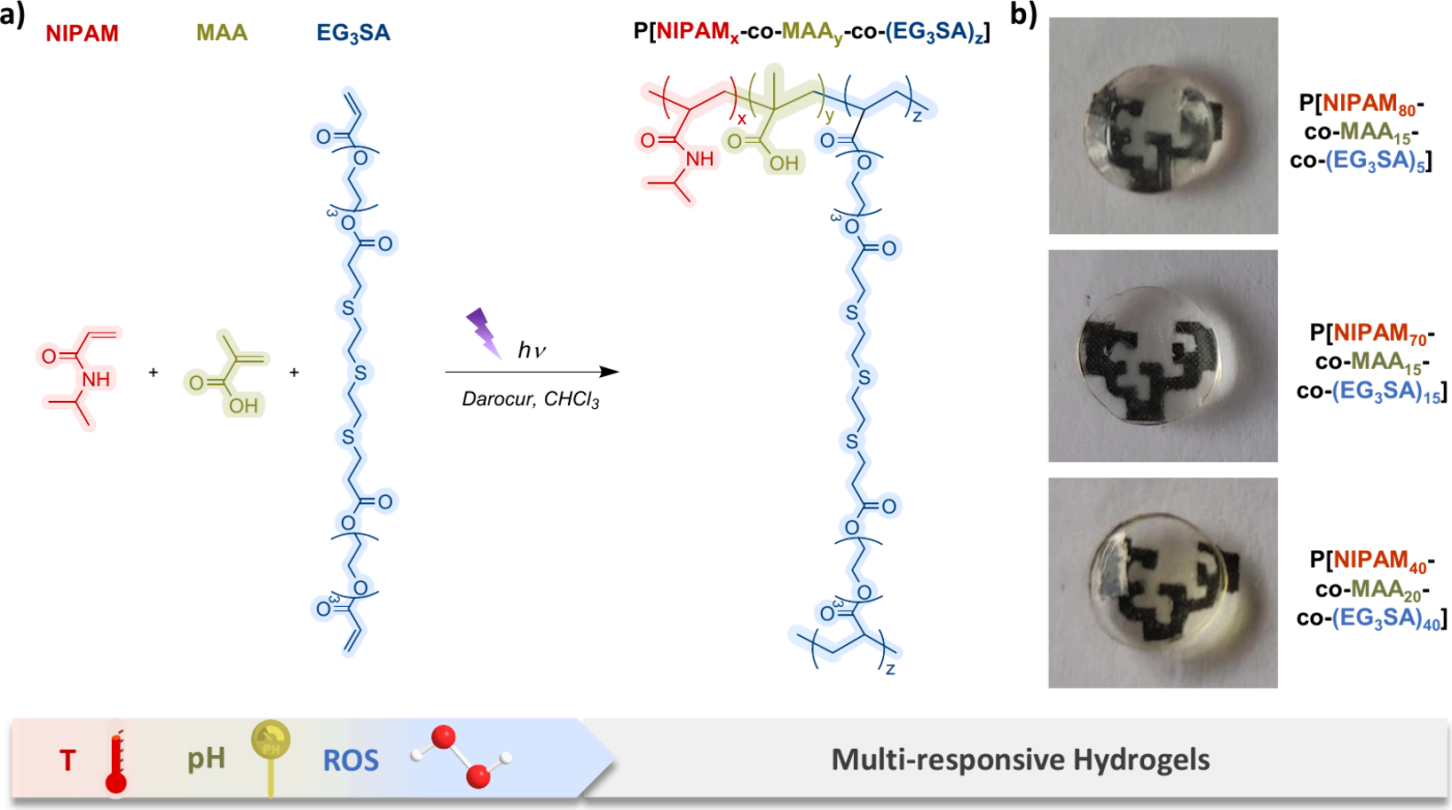
(a) Chemical route employed for the synthesis of P[NIPAM_*x*_-*co*-MAA_*y*_-*co*-(EG_3_SA)_*z*_] hydrogels by UV light at 365 nm for 3–5 min and using Darocur as a photoinitiator. (b) Pictures of the synthesized hydrogels with different monomer ratios.

The chemical characterization of the hydrogels was performed by infrared spectroscopy (Figure S1). The peak at 1720 cm^–1^ is attributed to C=O vibrations of the acid carbonyl groups of MAA and the acrylate groups of EG_3_SA. The peaks at 1650, 1540, and 1130 cm^–1^ are assigned to C=O, N—H, and C—N stretching of amide groups present in NIPAM. The peak at around 1455 cm^–1^ is attributed to C—H bending in the −(CH_3_)_2_ and −CH_2_ groups of NIPAM and MAA,^31^ and the peaks at 690 and 715 cm^–1^ are the signatures of symmetric and asymmetric dimethyl sulfide bonds, respectively. After H_2_O_2_ treatment, thioether groups of EG_3_SA are oxidized into sulfoxides and/or sulfones, as corroborated by the appearance of two peaks at 1020 and 1320 cm^–1^ corresponding to the stretching of the double bond S=O in sulfoxides and O=S=O in sulfones, respectively.^28^

The response of the hydrogels to different stimuli, ROS, temperature, and pH, was tested (Figure 2a). Four different scenarios can be considered. (i) Single pH-response: under nonoxidative conditions (PBS) at room temperature (25 °C < LCST of PNIPAM) and different pHs, swelling is controlled by the protonation state of MAA (Figure 2b). P[NIPAM_80_-*co*-MAA_15_-*co*-(EG_3_SA)_5_] hydrogels exhibited a ≈140 wt % swelling at pH 7.4. Increasing the EG_3_SA percentage resulted in cross-linking points and more reticulated hydrogels with less water-holding capacity decreasing the swelling of P[NIPAM_40_-*co*-MAA_20_-*co*-(EG_3_SA)_40_] up to ≈10%wt. At acidic pHs (<p*K*_a_ ≈ 5.5 of PMAA), carboxylic groups of PMAA are protonated (COOH) and hydrogels shrank, decreasing the swelling. For P[NIPAM_80_-*co*-MAA_15_-*co*-(EG_3_SA)_5_] hydrogels, a decrease of up to ≈47 wt % at pH 3 was observed, while swelling was almost negligible for P[NIPAM_40_-*co*-MAA_20_-*co*-(EG_3_SA)_40_]. At alkaline pH 11, COO^–^ deprotonates, promoting the swelling of P[NIPAM_80_-*co*-MAA_15_-*co*-(EG_3_SA)_5_] hydrogels up to ≈215%wt. (ii) Dual thermo- and pH-response under nonoxidative conditions (PBS) at 37 °C (>LCST of PNIPAM) and different pHs (Figure 2c): At this temperature, hydrogels contract due to the presence of PNIPAM. The swelling of P[NIPAM_80_-*co*-MAA_15_-*co*-(EG_3_SA)_5_] hydrogels decreased up to ≈93 wt % at pH 7.4 and ≈17 wt % at pH 3, whereas for P[NIPAM_70_-*co*-MAA_15_-*co*-(EG_3_SA)_15_] and P[NIPAM_40_-*co*-MAA_20_-*co*-(EG_3_SA)_40_] hydrogels with a larger content of EG_3_SA, the high cross-linking degree induced similar swelling values to those observed at 25 °C. The same tendency was observed at pH 11 (>p*K*_a_ of PMAA). (iii) Dual ROS- and pH-response under oxidative conditions (9 mM H_2_O_2_) at 25 °C and different pHs (Figure 2d): P[NIPAM_80_-*co*-MAA_15_-*co*-(EG_3_SA)_5_] hydrogels experienced a huge swelling of ≈465 wt % at pH 7.4 due to the oxidation of the thioether groups present in the EG_3_SA domains, which led to more hydrophilic hydrogels. This effect was less pronounced in hydrogels with a larger content of EG_3_SA due to their higher cross-linking density. All hydrogels exhibited the highest swelling properties in H_2_O_2_ at pH 11, as all copolymer components are in the most hydrophilic state, holding the largest quantity of water, ≈1320 wt % for P[NIPAM_80_-*co*-MAA_15_-*co*-(EG_3_SA)_5_], ≈615 wt % for P[NIPAM_70_-*co*-MAA_15_-*co*-(EG_3_SA)_15_], and ≈265 wt % for P[NIPAM_40_-*co*-MAA_20_-*co*-(EG_3_SA)_40_]. (iv) Triple ROS-, thermo-, and pH-response under oxidative conditions (9 mM H_2_O_2_) at 37 °C and different pHs, where all monomers are involved in the stimuli-responsive properties (Figure 2e): The least cross-linked P[NIPAM_80_-*co*-MAA_15_-*co*-(EG_3_SA)_5_] hydrogels presented the highest swelling at all pHs (Figures 1f,g and S2). At pH 7.4, P[NIPAM_80_-*co*-MAA_15_-*co*-(EG_3_SA)_5_] hydrogels showed the lowest swelling (≈93 wt %), as they contained the highest percentage of PNIPAM. The swelling decreased with the pH up to ≈8 wt % at pH 3 due to the combination of the shrinking behavior of PNIPAM and PMAA that camouflaged the hydrophilic oxidation properties of PEG_3_SA. At alkaline pH 11, the swelling increased exponentially due to the deprotonation of COO^–^ groups of PMAA together with the more hydrophilic oxidized PEG_3_SA, which led to the hydrogel’s breaking, probably due to the high pressure produced by the water, which broke their network and made them difficult to handle.

**Figure 2:**
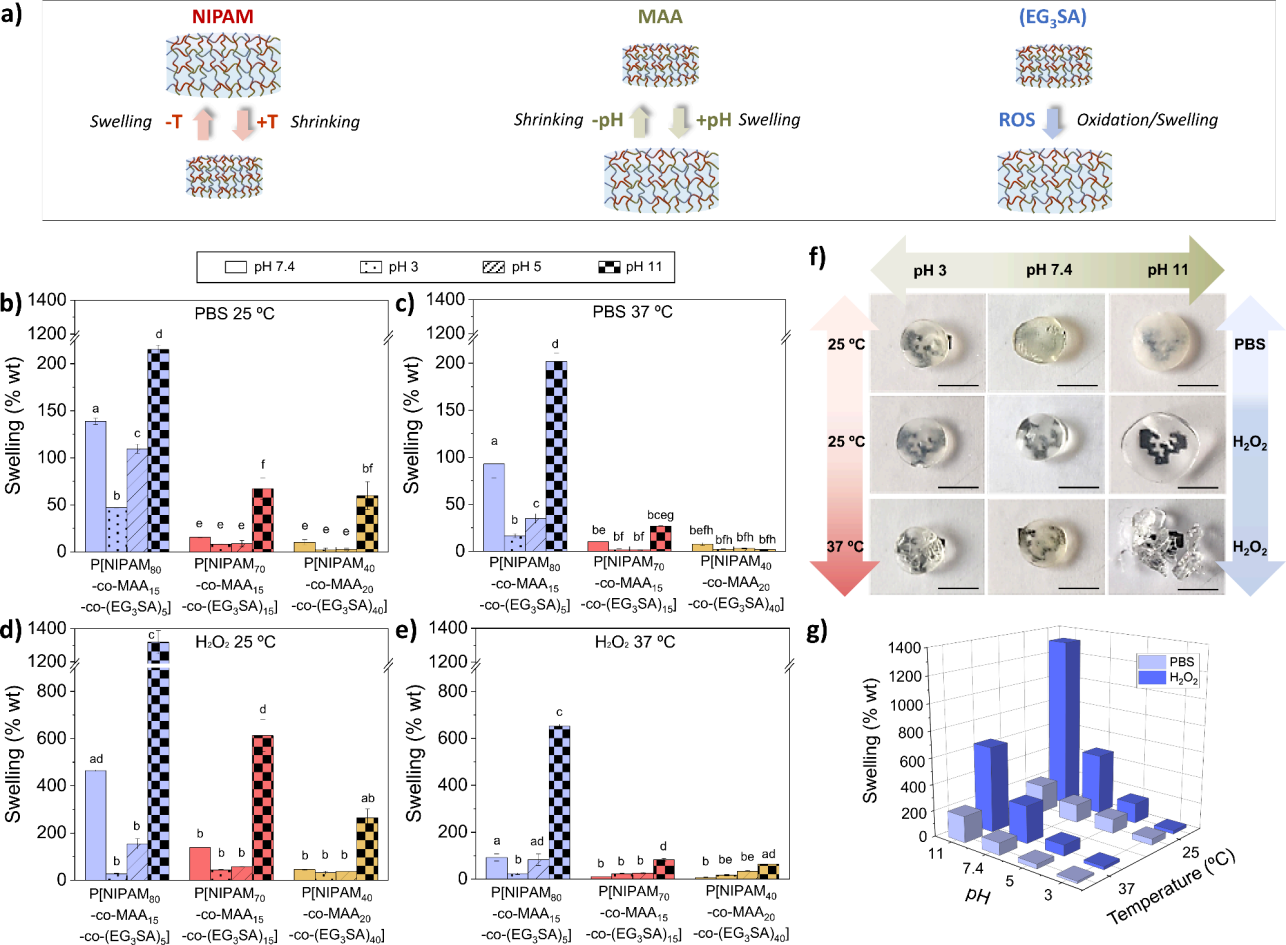
(a) Schematic representation of the swelling/shrinking behavior of each monomer within the P[NIPAM_*x*_-*co*-MAA_*y*_-*co*-(EG_3_SA)_*z*_] hydrogels in response to external stimuli, temperature, pH, and/or ROS. (b) Swelling of P[NIPAM_*x*_-*co*-MAA_*y*_-*co*-(EG_3_SA)_*z*_] hydrogels at different pHs immersed in (b) PBS at 25 °C, (c) PBS at 37 °C, (d) 9 mM H_2_O_2_ at 25 °C, and (e) 9 mM H_2_O_2_ at 37 °C, for 24 h. Diagrams (b)–(e) include the mean and standard deviation (*n* = 3) and the ANOVA results. Different letters indicate statistically significant differences at a significance level of *p* < 0.05 using Tukey’s test. Bars with no common letters are significantly different (*p* < 0.05). (f) Representative pictures of P[NIPAM_80_-*co*-MAA_15_-*co*-(EG_3_SA)_5_] hydrogels after 24 h at different temperatures, pHs, and under nonoxidant (PBS) or oxidant (H_2_O_2_) conditions. Scale bars = 5 mm. (g) Swelling comparison of P[NIPAM_80_-*co*-MAA_15_-*co*-(EG_3_SA)_5_] hydrogels at different temperatures (25 and 37 °C) and pHs (3, 5, 7.4 and 11), in Phosphate Buffer Solution (PBS) or H_2_O_2_.

P[NIPAM_*n*_-*co*-MAA_*m*_-*co*-(EG_3_SA)_*x*_] hydrogels were tested as scaffolds to encapsulate an anti-inflammatory drug, ketoprofen (KET). The KET release under different stimuli (temperature, pH, ROS; Figure 3a,b) was correlated with the hydrogels’ swelling behavior (Figure 2). P[NIPAM_80_-*co*-MAA_15_-*co*-(EG_3_SA)_5_] and P[NIPAM_70_-*co*-MAA_15_-*co*-(EG_3_SA)_15_] hydrogels showed a much higher capability of releasing KET than P[NIPAM_40_-*co*-MAA_20_-*co*-(EG_3_SA)_40_], in agreement with swelling tests. P[NIPAM_80_-*co*-MAA_15_-*co*-(EG_3_SA)_5_] hydrogels released 0.26 mg/mL of KET after 24 h in PBS at pH 7.4 and 25 °C. The release decreased up to 0.21 mg/mL at 37 °C due to the NIPAM-induced shrinking trapping a higher part of KET molecules inside. The decrease of pH to 5, at 37 °C, induced a slight reduction of the KET released up to 0.19 mg/mL because of the MAA-induced shrinking, while the pH increase to 11 increased the release (0.33 mg/mL). In the presence of H_2_O_2_ at pH 7.4 and 37 °C, the release of KET increased (0.27 mg/mL) due to the oxidation of the thioether groups of EG_3_SA becoming more hydrophilic, while the decrease of pH slightly reduced the KET released (0.25 mg/mL). This hydrophilic effect is more evident over time as the release of KET increased after 72 h. P[NIPAM_70_-*co*-MAA_15_-*co*-(EG_3_SA)_15_] hydrogels showed a similar behavior. The same trends were observed in the case of P[NIPAM_40_-*co*-MAA_20_-*co*-(EG_3_SA)_40_] hydrogels, although the concentration of KET released was much lower due to their higher cross-linking degree and consequently lower swelling capacity. Therefore, all hydrogels were thermo-, pH-, and ROS- responsive leading to tunable KET release profiles.

**Figure 3:**
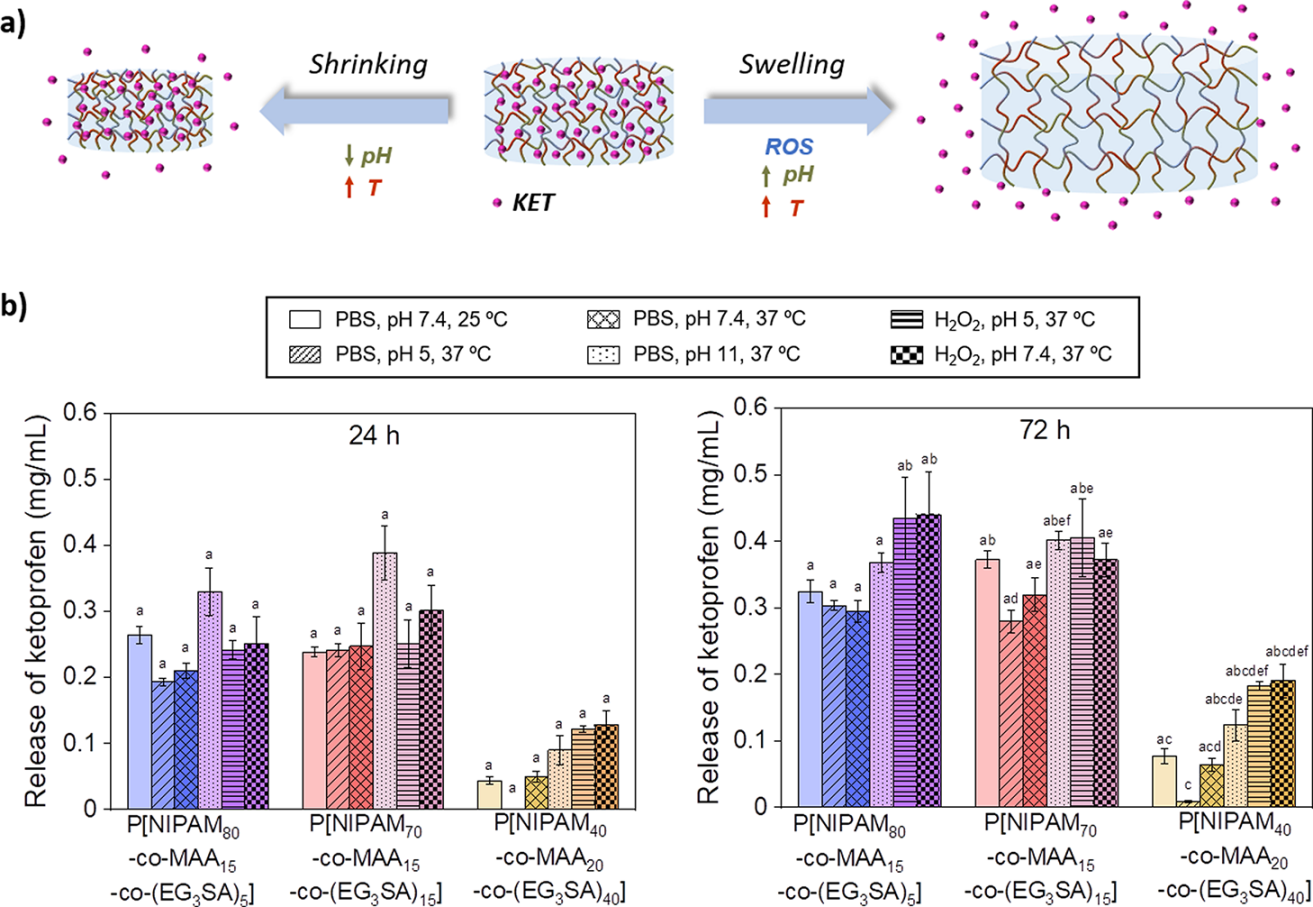
(a) Schematic representation of the ketoprofen (KET) release from P[NIPAM_*x*_-*co*-MAA_*y*_-*co*-(EG_3_SA)_*z*_] hydrogels under different conditions of temperature, pH, and ROS. (b) Release of KET from P[NIPAM_*x*_-*co*-MAA_*y*_-*co*-(EG_3_SA)_*z*_] hydrogels under nonoxidative conditions in PBS, and oxidative conditions in the presence of 9 mM H_2_O_2_, at 25 or 37 °C, and pH 5, 7.4, or 11 for 24 and 72 h. Diagrams include the mean and standard deviation (*n* = 3) and the ANOVA results. Different letters indicate statistically significant differences at a significance level of *p* < 0.05 using Tukey’s test. Bars with no common letters are significantly different (*p* < 0.05).

The additive manufacturing by DLP 3D printing was initially studied by photorheology (Figure 4a). Before irradiation (0–60 s), the loss modulus (*G*′′) was higher than the storage modulus (*G*′), pointing out the liquid-like state of the copolymer inks. After 60 s, the UV light was switched and the photopolymerization process started reaching a solid-like state (*G*′ > *G*′′) in a few seconds. The photopolymerization time decreased with an increase in the EG_3_SA concentration from 20 s for P[NIPAM_80_-*co*-MAA_15_-*co*-(EG_3_SA)_5_] to 12 and 10 s for P[NIPAM_70_-*co*-MAA_15_-*co*-(EG_3_SA)_15_] and P[NIPAM_40_-*co*-MAA_20_-*co*-(EG_3_SA)_40_], respectively. As no significant differences were observed between hydrogels with 5% and 15% mol EG_3_SA, only hydrogels with the lowest and highest percentage of this monomer, 5% and 40% mol, were studied in further experiments. The copolymers with 5% and 40% mol EG_3_SA were successfully processed by DLP 3D printing to fabricate customized multihollow scaffolds (Figure 4b). Thanks to their stimuli-responsive properties, they became 4D-printable hydrogels. The printing resolution increased with the percentage of EG_3_SA monomer within the copolymers, but the hydrogels were more brittle. The mechanical properties in the presence of different stimuli were characterized by rheology. In all cases, *G*′ was higher than *G*′′, corroborating the hydrogel formation (Figure 4c). Three different conditions were tested to characterize the hydrogels based on the most representative biological conditions: (i) Physiological mimicking conditions (PBS, pH 7.4, 37 °C): *G*′ increased with NIPAM percentage within the hydrogels, from ≈2 × 10^4^ Pa for P[NIPAM_40_-*co*-MAA_20_-*co*-(EG_3_SA)_40_] (Figure S3) to ≈6 × 10^4^ Pa for P[NIPAM_80_-*co*-MAA_15_-*co*-(EG_3_SA)_5_] due to their higher contraction at physiological temperature making them less flexible. (ii) Oxidation in the presence of ROS (H_2_O_2_), which are present in inflammatory diseases,^32^ at pH 7.4 and 37 °C (Figure 4d). The mechanical properties were highly influenced by the thermoresponse of NIPAM and ROS-response of EG_3_SA. *G*′ increased up to ≈1 × 10^5^ Pa in P[NIPAM_40_-*co*-MAA_20_-*co*-(EG_3_SA)_40_] hydrogels due to the higher percentage of EG_3_SA that led to the formation of sulfoxides and sulfones, thus, allowing them to hold a high quantity of water and making them more brittle. (iii) Oxidation (H_2_O_2_) at pH 5 and 37 °C (Figure 4e). No significant differences were observed in *G*′ of P[NIPAM_40_-*co*-MAA_20_-*co*-(EG_3_SA)_40_] hydrogels between pH 7.4 and pH 5, probably because they reached the maximum swelling capacity before breaking. *G*′ of P[NIPAM_80_-*co*-MAA_15_-*co*-(EG_3_SA)_5_] hydrogels decreased to ≈3 × 10^4^ Pa because of the less amount of cross-linker EG_3_SA and the higher elasticity of the oxidized chains.

**Figure 4:**
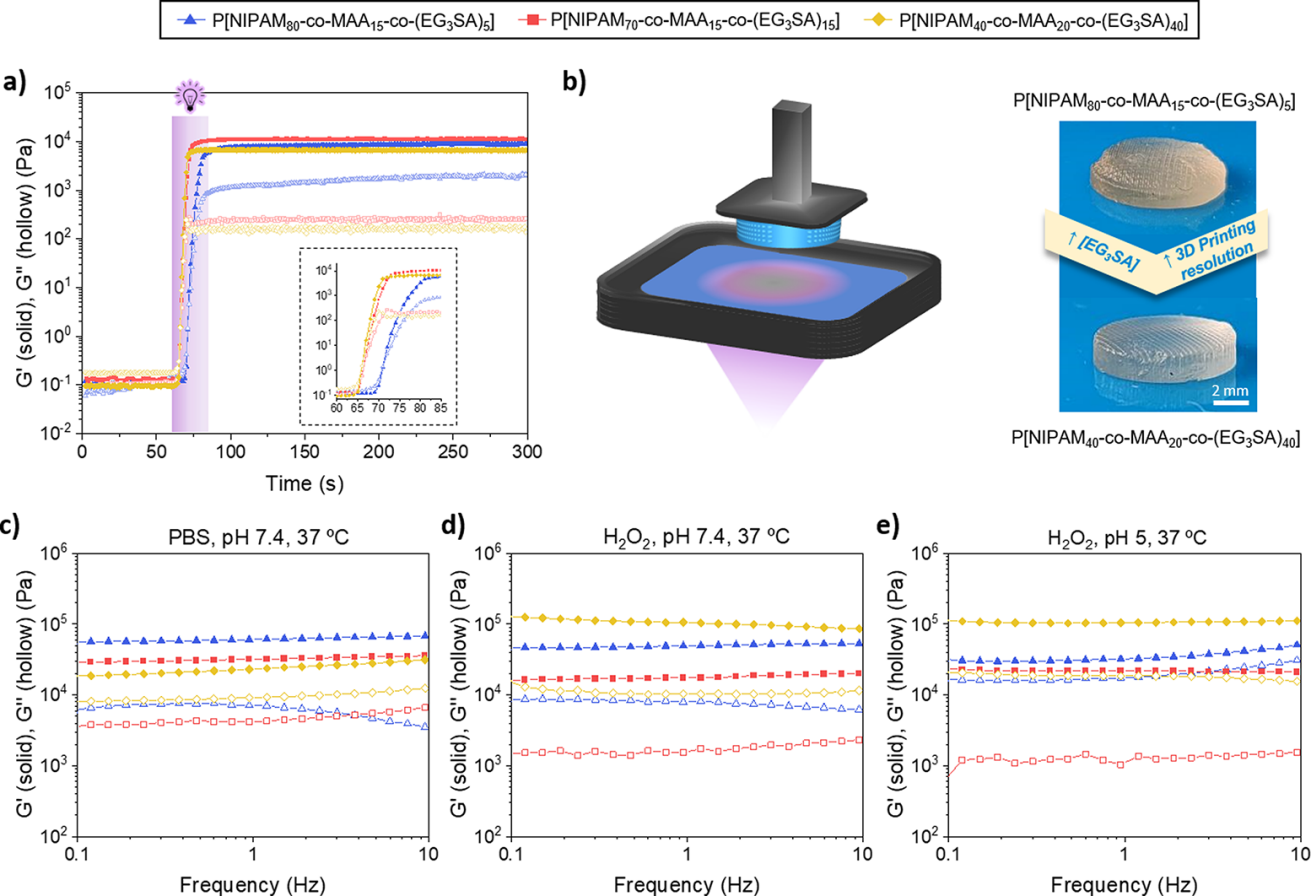
(a) Photorheological properties to determine the gel point (*G*′ > *G*′′) when the inks are irradiated for 10–20 s (from 60 s), leading to P[NIPAM_*x*_-*co*-MAA_*y*_-*co*-(EG_3_SA)_*z*_] hydrogels. (b) Schematic representation of the DLP process employed to print the hydrogels (left), and shape-defined 4D-printed P[NIPAM_*x*_-*co*-MAA_*y*_-*co*-(EG_3_SA)_*z*_] hydrogel scaffolds with pores (right). Rheological properties of P[NIPAM_*x*_-*co*-MAA_*y*_-*co*-(EG_3_SA)_*z*_] hydrogels in the presence of different stimuli: (c) PBS, pH 7.4, 37 °C, (d) H_2_O_2_, pH 7.4, 37 °C, and (e) H_2_O_2_, pH 5, 37 °C.

The cytotoxicity of nonloaded and KET-loaded P[NIPAM_*x*_-*co*-MAA_*y*_-*co*-(EG_3_SA)_*z*_] hydrogels was tested *in vitro* with mouse embryonic fibroblasts (NIH 3T3) and RAW 264.7 murine macrophage (RAW) cells. These cell lines were selected as representative models involved in inflammatory processes during tissue repair,^33−36^ in which macrophages modulate inflammation and fibroblasts lay down a new extracellular matrix. P[NIPAM_80_-*co*-MAA_15_-*co*-(EG_3_SA)_5_] hydrogels were not cytotoxic, showing NIH 3T3 and RAW cell viabilities higher than 90% (Figure 5a,b). However, P[NIPAM_40_-*co*-MAA_20_-*co*-(EG_3_SA)_40_] hydrogels reduced the viability of NIH 3T3 (≈ 85%) and RAW (≈ 65%) cells and were discarded for the next experiments. It was observed that the presence of the EG_3_SA within P[NIPAM_80_-*co*-MAA_15_-*co*-(EG_3_SA)_5_] hydrogels favored the NIH 3T3 cell adhesion in comparison with P[NIPAM_90_-*co*-MAA_10_] hydrogels (Figure 5c,d). NIH 3T3 cell morphology was visualized by staining cell nuclei with Hoechst (blue staining) and cytoskeleton (F-actin fibers) with phalloidin-rhodamine (orange staining). On P[NIPAM_80_-*co*-MAA_15_-*co*-(EG_3_SA)_5_] hydrogels, NIH 3T3 cells showed an elongated morphology and cell spreading, which was not observed in P[NIPAM_90_-*co*-MAA_10_] hydrogels where fibroblasts exhibited a round morphology forming clusters. It is known that cell adhesion is influenced by chemical groups present on the surface of the hydrogels^37^ and specifically enhanced by sulfonic groups,^38^ which induce a reorganization of the actin cytoskeleton of fibroblasts.^39,40^ Thus, the thioether groups present on the EG_3_SA domains favored the cell adhesion on P[NIPAM_80_-*co*-MAA_15_-*co*-(EG_3_SA)_5_] hydrogels. The anti-inflammatory properties of nonloaded and KET-loaded P[NIPAM_80_-*co*-MAA_15_-*co*-(EG_3_SA)_5_] hydrogels were tested in contact with RAW cells, which can polarize to their pro-inflammatory phenotype (M1) when they are activated by lipopolysaccharide (LPS) and start to overproduce nitric oxide (NO). The anti-inflammatory capacity of the hydrogels was determined by measuring the NO production of LPS-activated RAW cells (LPS-RAW) seeded on the hydrogels for 24 h (Figure 5e) in comparison with LPS-RAW cells seeded on the well plate (positive control, inflammatory conditions untreated - ICU) and non-LPS-activated RAW cells seeded on the hydrogels (negative control, noninflammatory conditions - NIC). The NO released by LPS-RAW cells seeded on top of P[NIPAM_80_-*co*-MAA_15_-*co*-(EG_3_SA)_5_] hydrogels decreased up to 29.9 ± 2.6% after 24 h, reaching the basal value of non-LPS-activated RAW cells on the hydrogels (NIC = 32.3 ± 1.1%). Nonsignificant differences were detected in comparison with KET-loaded P[NIPAM_80_-*co*-MAA_15_-*co*-(EG_3_SA)_5_] hydrogels that also decreased the NO production (30.1 ± 4.4%) up to basal values. To quantify the anti-inflammatory capacity of P[NIPAM_80_-*co*-MAA_15_-*co*-(EG_3_SA)_5_] hydrogels, NO values were compared with those of LPS-RAW cells seeded on a plate and brought in contact with different ketoprofen concentrations (Figure S4). Results showed that NO released by LPS-RAW cells decreased up to basal values from 0.3 mg/mL KET approximately. Overall, these results proved the excellent anti-inflammatory capacity of P[NIPAM_80_-*co*-MAA_15_-*co*-(EG_3_SA)_5_] hydrogels, *per se*, without encapsulating an anti-inflammatory drug (Figure 5e). At the initial stage, RAW cells are seeded on top of P[NIPAM_80_-*co*-MAA_15_-*co*-(EG_3_SA)_5_] hydrogels. They are in a noninflammatory stage and NO production is at the basal value. In the second stage, the inflammatory process is induced by activation of RAW cells with LPS and they start to overproduce NO (a type of ROS). Then, in the last stage, the ROS produced by LPS-RAW cells are trapped by the P[NIPAM_80_-*co*-MAA_15_-*co*-(EG_3_SOA)_5_] hydrogels in the oxidized EG_3_SA domains formed by sulfoxides and sulfones (EG_3_SOA), thus reducing the inflammatory process and the NO production by RAW cells up to basal values of noninflammatory conditions. This interesting achievement opens the route for the fabrication of 4D printable anti-inflammatory scaffolds in a customized manner with anti-inflammatory properties.

**Figure 5:**
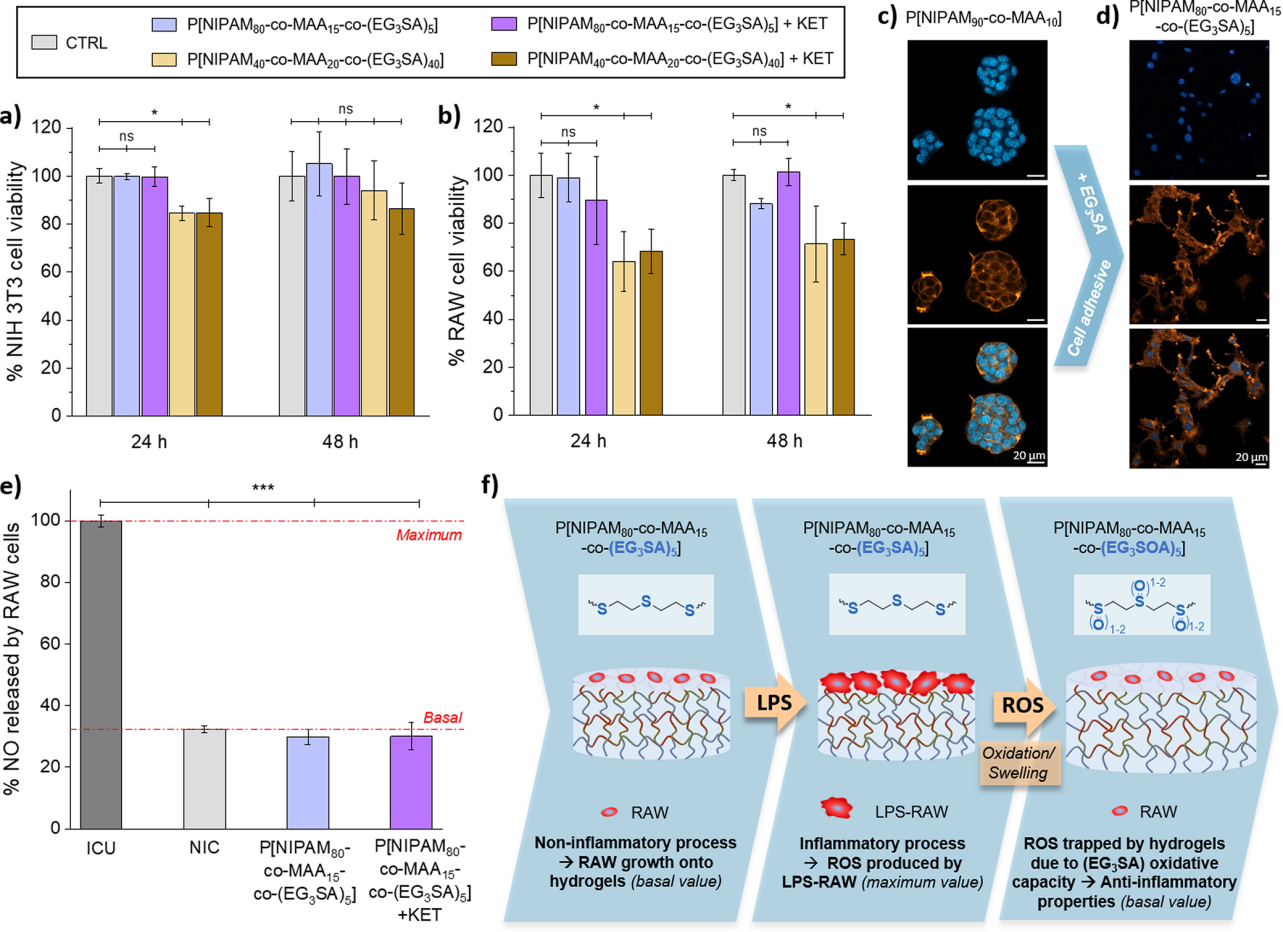
In vitro cytotoxicity tests of nonloaded and KET-loaded P[NIPAM_*x*_-*co*-MAA_*y*_-*co*-(EG_3_SA)_*z*_] hydrogels in contact with (a) NIH 3T3 and (b) RAW cells for 24 and 48 h. (c) NIH 3T3 cell adhesion on (c) P[NIPAM_90_-*co*-MAA_10_] and (d) P[NIPAM_80_-*co*-MAA_15_-*co*-(EG_3_SA)_5_] hydrogels. (e) Nitric oxide (NO) released by LPS-RAW cells seeded on the plate (ICU), non-LPS-RAW cells seeded on the plate (NIC), and LPS-RAW cells seeded on the hydrogels. Diagrams (a), (b), and (e) include the mean and standard deviation (*n* = 3) and the ANOVA results at significance levels of *p < 0.05 and ***p < 0.001 using Tukey’s test (ns means nonstatistically significant differences). (f) Schematic representation of the inflammatory process induced by the activation of RAW cells, growth on top of the hydrogels, with LPS leading to ROS production, and the anti-inflammatory properties of P[NIPAM_80_-*co*-MAA_15_-*co*-(EG_3_SA)_5_] hydrogels, which trapped ROS due to the oxidative capacity of the (EG_3_SA) block.

In conclusion, triple-responsive hydrogels were synthesized by photopolymerization of thermoresponsive NIPAM, pH-responsive MAA, and ROS-responsive EG_3_SA monomers. Thus, it also allowed their additive manufacturing by DLP leading to 4D printed shape-defined hydrogels. The swelling properties of P[NIPAM_*x*_-*co*-MAA_*y*_-*co*-(EG_3_SA)_*z*_] hydrogels decreased with the temperature increase from 25 to 37 °C, due to the contraction of PNIPAM chains above their LCST, and with the pH decrease from 7.4 to 3, because of the protonation of the carboxylic groups of PMAA below their p*K*_a_. On the contrary, swelling increased with the pH increase from 7.4 to 11, due to the deprotonation of carboxylic groups, and with the presence of ROS (i.e., H_2_O_2_) because of the oxidation of the thioether groups in EG_3_SA into sulfoxides and sulfones making them more hydrophilic. P[NIPAM_*x*_-*co*-MAA_*y*_-*co*-(EG_3_SA)_*z*_] hydrogels were used as carriers for the controlled release of ketoprofen. The mechanical properties of the hydrogels were characterized by rheology showing a variation of the initial *G*′ values (≈10^4^ Pa) that depended on the applied stimuli and could reach up to ≈10^5^ Pa. Cell tests pointed out P[NIPAM_80_-*co*-MAA_15_-*co*-(EG_3_SA)_5_] hydrogels presented the optimal stimuli-responsive performance while being noncytotoxic, at the same time they possessed anti-inflammatory properties *per se*.

## Supporting Information Available

Materials and methods, ^1^H NMR spectra of EG_3_SA monomer in CDCl_3_, FTIR spectra of P[NIPAM_70_-*co*-MAA_15_-*co*-(EG_3_SA)_15_] hydrogels at different conditions, representative pictures of P[NIPAM_*x*_-*co*-MAA_*y*_-*co*-(EG_3_SA)_*z*_] hydrogels under different conditions and swelling comparison, rheological properties of P[NIPAM_*x*_-*co*-MAA_*y*_-*co*-(EG_3_SA)_*z*_] hydrogels in PBS at pH 7.4 and 25 °C, and nitric oxide (NO) released by RAW cells in the presence of different ketoprofen concentrations (PDF)

## References

[ref1] Yu H.; Gao R.; Liu Y.; Fu L.; Zhou J.; Li L. Stimulus-Responsive Hydrogels as Drug Delivery Systems for Inflammation Targeted Therapy. Adv. Sci. 2024, 11 (1), 2306152 10.1002/advs.202306152. PMC10767459 37985923

[ref2] Carleton M. M.; Locke M.; Sefton M. V. Methacrylic acid-based hydrogels enhance skeletal muscle regeneration after volumetric muscle loss in mice. Biomaterials 2021, 275, 120909 10.1016/j.biomaterials.2021.120909. 34087582

[ref3] Tang L.; Wang L.; Yang X.; Feng Y.; Li Y.; Feng W. Poly(N-isopropylacrylamide)-based smart hydrogels: Design, properties and applications. Prog. Mater. Sci. 2021, 115, 100702 10.1016/j.pmatsci.2020.100702.

[ref4] Jo Y.-J.; Gulfam M.; Jo S.-H.; Gal Y.-S.; Oh C.-W.; Park S.-H.; Lim K. T. Multi-stimuli responsive hydrogels derived from hyaluronic acid for cancer therapy application. Carbohydr. Polym. 2022, 286, 119303 10.1016/j.carbpol.2022.119303. 35337532

[ref5] Qin H.; Zhang T.; Li N.; Cong H.-P.; Yu S.-H. Anisotropic and self-healing hydrogels with multi-responsive actuating capability. Nat. Commun. 2019, 10 (1), 2202 10.1038/s41467-019-10243-8. 31101823 PMC6525195

[ref6] Sigolaeva L. V.; Gladyr S. Y.; Gelissen A. P. H.; Mergel O.; Pergushov D. V.; Kurochkin I. N.; Plamper F. A.; Richtering W. Dual-Stimuli-Sensitive Microgels as a Tool for Stimulated Spongelike Adsorption of Biomaterials for Biosensor Applications. Biomacromolecules 2014, 15 (10), 3735–3745. 10.1021/bm5010349. 25211008

[ref7] Pourjavadi A.; Heydarpour R.; Tehrani Z. M. Multi-stimuli-responsive hydrogels and their medical applications. New J. Chem. 2021, 45 (35), 15705–15717. 10.1039/D1NJ02260A.

[ref8] Downs F. G.; Lunn D. J.; Booth M. J.; Sauer J. B.; Ramsay W. J.; Klemperer R. G.; Hawker C. J.; Bayley H. Multi-responsive hydrogel structures from patterned droplet networks. Nat. Chem. 2020, 12 (4), 363–371. 10.1038/s41557-020-0444-1. 32221498 PMC7117959

[ref9] Peñas-Núñez S. J.; Mecerreyes D.; Criado-Gonzalez M. Recent Advances and Developments in Injectable Conductive Polymer Gels for Bioelectronics. ACS Appl. Bio Mater. 2024, na 10.1021/acsabm.3c01224. PMC11653406 38364213

[ref10] Tran H. B. D.; Vazquez-Martel C.; Catt S. O.; Jia Y.; Tsotsalas M.; Spiegel C. A.; Blasco E. 4D Printing of Adaptable “Living” Materials Based on Alkoxyamine Chemistry. Adv. Funct. Mater. 2024, 34, 2315238 10.1002/adfm.202315238.

[ref11] Spiegel C. A.; Hackner M.; Bothe V. P.; Spatz J. P.; Blasco E. 4D Printing of Shape Memory Polymers: From Macro to Micro. Adv. Funct. Mater. 2022, 32 (51), 2110580 10.1002/adfm.202110580.

[ref12] Matsumoto N. M.; Buchman G. W.; Rome L. H.; Maynard H. D. Dual pH- and temperature-responsive protein nanoparticles. Eur. Polym. J. 2015, 69, 532–539. 10.1016/j.eurpolymj.2015.01.043. 26365998 PMC4565796

[ref13] Gao Y.; Wei M.; Li X.; Xu W.; Ahiabu A.; Perdiz J.; Liu Z.; Serpe M. J. Stimuli-responsive polymers: Fundamental considerations and applications. Macromol. Res. 2017, 25 (6), 513–527. 10.1007/s13233-017-5088-7.

[ref14] Reineke T. M. Stimuli-Responsive Polymers for Biological Detection and Delivery. ACS Macro Lett. 2016, 5 (1), 14–18. 10.1021/acsmacrolett.5b00862. 35668593

[ref15] Beck J. B.; Rowan S. J. Multistimuli, Multiresponsive Metallo-Supramolecular Polymers. J. Am. Chem. Soc. 2003, 125 (46), 13922–13923. 10.1021/ja038521k. 14611204

[ref16] Cudjoe E.; Khani S.; Way A. E.; Hore M. J. A.; Maia J.; Rowan S. J. Biomimetic Reversible Heat-Stiffening Polymer Nanocomposites. ACS Cent. Sci. 2017, 3 (8), 886–894. 10.1021/acscentsci.7b00215. 28852703 PMC5571458

[ref17] Robinson D. N.; Peppas N. A. Preparation and Characterization of pH-Responsive Poly(methacrylic acid-g-ethylene glycol) Nanospheres. Macromolecules 2002, 35 (9), 3668–3674. 10.1021/ma011525u.

[ref18] Gao X.; Cao Y.; Song X.; Zhang Z.; Xiao C.; He C.; Chen X. pH- and thermo-responsive poly(N-isopropylacrylamide-co-acrylic acid derivative) copolymers and hydrogels with LCST dependent on pH and alkyl side groups. J. Mater. Chem. B 2013, 1 (41), 5578–5587. 10.1039/c3tb20901f. 32261182

[ref19] Belman-Flores C. E.; Herrera-Kao W.; Vargas-Coronado R. F.; May-Pat A.; Oliva A. I.; Rodríguez-Fuentes N.; Vázquez-Torres H.; Cauich-Rodríguez J. V.; Cervantes-Uc J. M. Synthesis and characterization of pH sensitive hydrogel nanoparticles based on poly(*N*-isopropyl acrylamide-*co*-methacrylic acid). J. Mater. Sci.: Mater. Med. 2020, 31 (8), 61 10.1007/s10856-020-06400-x. 32696259

[ref20] Han Z.; Wang P.; Mao G.; Yin T.; Zhong D.; Yiming B.; Hu X.; Jia Z.; Nian G.; Qu S.; Yang W. Dual pH-Responsive Hydrogel Actuator for Lipophilic Drug Delivery. ACS Appl. Mater. Interfaces 2020, 12 (10), 12010–12017. 10.1021/acsami.9b21713. 32053341

[ref21] Zhao Y.; Shi C.; Yang X.; Shen B.; Sun Y.; Chen Y.; Xu X.; Sun H.; Yu K.; Yang B.; Lin Q. pH- and Temperature-Sensitive Hydrogel Nanoparticles with Dual Photoluminescence for Bioprobes. ACS Nano 2016, 10 (6), 5856–5863. 10.1021/acsnano.6b00770. 27232534

[ref22] Sies H.; Jones D. P. Reactive oxygen species (ROS) as pleiotropic physiological signalling agents. Nat. Rev. Mol. Cell Biol. 2020, 21 (7), 363–383. 10.1038/s41580-020-0230-3. 32231263

[ref23] Shields H. J.; Traa A.; Van Raamsdonk J. M. Beneficial and Detrimental Effects of Reactive Oxygen Species on Lifespan: A Comprehensive Review of Comparative and Experimental Studies. Front. Cell Dev. Biol. 2021, 9, 628157 10.3389/fcell.2021.628157. 33644065 PMC7905231

[ref24] Xu Q.; He C.; Xiao C.; Chen X. Reactive Oxygen Species (ROS) Responsive Polymers for Biomedical Applications. Macromol. Biosci. 2016, 16 (5), 635–646. 10.1002/mabi.201500440. 26891447

[ref25] Criado-Gonzalez M.; Mecerreyes D. Thioether-based ROS responsive polymers for biomedical applications. J. Mater. Chem. B 2022, 10 (37), 7206–7221. 10.1039/D2TB00615D. 35611805

[ref26] Napoli A.; Valentini M.; Tirelli N.; Müller M.; Hubbell J. A. Oxidation-responsive polymeric vesicles. Nat. Mater. 2004, 3 (3), 183–189. 10.1038/nmat1081. 14991021

[ref27] Yan B.; Zhang Y.; Wei C.; Xu Y. Facile synthesis of ROS-responsive biodegradable main chain poly(carbonate-thioether) copolymers. Polym. Chem. 2018, 9 (7), 904–911. 10.1039/C7PY01908D.

[ref28] Regato-Herbella M.; Morhenn I.; Mantione D.; Pascuzzi G.; Gallastegui A.; Caribé dos Santos Valle A. B.; Moya S. E.; Criado-Gonzalez M.; Mecerreyes D. ROS-Responsive 4D Printable Acrylic Thioether-Based Hydrogels for Smart Drug Release. Chem. Mater. 2024, 36 (3), 1262–1272. 10.1021/acs.chemmater.3c02264. 38370279 PMC10870821

[ref29] Liu J.; Li Y.; Chen S.; Lin Y.; Lai H.; Chen B.; Chen T. Biomedical Application of Reactive Oxygen Species-Responsive Nanocarriers in Cancer, Inflammation, and Neurodegenerative Diseases. Front. Chem. 2020, 8, 838 10.3389/fchem.2020.00838. 33062637 PMC7530259

[ref30] Zhang R.; Liu R.; Liu C.; Pan L.; Qi Y.; Cheng J.; Guo J.; Jia Y.; Ding J.; Zhang J.; Hu H. A pH/ROS dual-responsive and targeting nanotherapy for vascular inflammatory diseases. Biomaterials 2020, 230, 119605 10.1016/j.biomaterials.2019.119605. 31740099

[ref31] Pardeshi P. M.; Mungray A. A. Photo-polymerization as a new approach to fabricate the active layer of forward osmosis membrane. Sci. Rep. 2019, 9 (1), 1937 10.1038/s41598-018-36346-8. 30760728 PMC6374509

[ref32] Khan A. Q.; Agha M. V.; Sheikhan K. S. A. M.; Younis S. M.; Tamimi M. A.; Alam M.; Ahmad A.; Uddin S.; Buddenkotte J.; Steinhoff M. Targeting deregulated oxidative stress in skin inflammatory diseases: An update on clinical importance. Biomed. Pharmacother. 2022, 154, 113601 10.1016/j.biopha.2022.113601. 36049315

[ref33] Mescher A. L. Macrophages and fibroblasts during inflammation and tissue repair in models of organ regeneration. Regeneration 2017, 4 (2), 39–53. 10.1002/reg2.77. 28616244 PMC5469729

[ref34] Buechler M. B.; Fu W.; Turley S. J. Fibroblast-macrophage reciprocal interactions in health, fibrosis, and cancer. Immunity 2021, 54 (5), 903–915. 10.1016/j.immuni.2021.04.021. 33979587

[ref35] Witherel C. E.; Abebayehu D.; Barker T. H.; Spiller K. L. Macrophage and Fibroblast Interactions in Biomaterial-Mediated Fibrosis. Adv. Healthc. Mater. 2019, 8 (4), 1801451 10.1002/adhm.201801451. PMC6415913 30658015

[ref36] Criado-Gonzalez M.; Espinosa-Cano E.; Rojo L.; Boulmedais F.; Aguilar M. R.; Hernández R. Injectable Tripeptide/Polymer Nanoparticles Supramolecular Hydrogel: A Candidate for the Treatment of Inflammatory Pathologies. ACS Appl. Mater. Interfaces 2022, 14 (8), 10068–10080. 10.1021/acsami.1c22993. 35179869

[ref37] Tallawi M.; Rosellini E.; Barbani N.; Cascone M. G.; Rai R.; Saint-Pierre G.; Boccaccini A. R. Strategies for the chemical and biological functionalization of scaffolds for cardiac tissue engineering: a review. J. R. Soc. Interface. 2015, 12 (108), 20150254 10.1098/rsif.2015.0254. 26109634 PMC4528590

[ref38] Kolluru G. K.; Shen X.; Kevil C. G. Reactive Sulfur Species. Arterioscler. Thromb. Vasc. Biol. 2020, 40 (4), 874–884. 10.1161/ATVBAHA.120.314084. 32131614 PMC7098439

[ref39] Kowalczyńska H. M.; Inkielman M.; Nowak-Wyrzykowska M.; Stołowska L.; Doroszewski J. Interaction of L1210 cells with sulfonated polystyrene in the absence of serum: adhesion and three-dimensional cell shape. Colloids Surf. B Biointerfaces 2003, 30 (3), 193–206. 10.1016/S0927-7765(03)00086-9.

[ref40] Wilson C. G.; Sisco P. N.; Gadala-Maria F. A.; Murphy C. J.; Goldsmith E. C. Polyelectrolyte-coated gold nanorods and their interactions with type I collagen. Biomaterials 2009, 30 (29), 5639–5648. 10.1016/j.biomaterials.2009.07.011. 19646751 PMC2754819

