# Supplementary material for: Multiresponsive 4D Printable Hydrogels with Anti-Inflammatory Properties
Source: ACS Macro Lett. 2024 Aug 14;13(9):1119–26. doi: 10.1021/acsmacrolett.4c00404 (PMC11411719; doi:10.1021/acsmacrolett.4c00404)
Supplement: Supplementary file 1 — mz4c00404_si_001.pdf [file mz4c00404_si_001.pdf]

SUPPORTING INFORMATION

Multi-responsive 4D printable hydrogels with anti-inflammatory properties

Maria Regato-Herbella,[1,2] *Daniele Mantione,*[1,3] *Agustín Blachman,*[4] *Antonela Gallastegui,*[1] *Graciela C. Calabrese,*[4] *Sergio E. Moya,*[2*] *David Mecerreyes*[1,3*], *Miryam Criado-Gonzalez*[1*]

[1] POLYMAT University of the Basque Country UPV/EHU, Joxe Mari Korta Center. Avda. Tolosa 72, 20018, Donostia-San Sebastián, Spain

[2] Center for Cooperative Research in Biomaterials (CIC biomaGUNE), Basque Research and Technology Alliance (BRTA). Paseo de Miramón 194, 20014, Donostia-San Sebastián, Spain.

[3] Ikerbasque, Basque Foundation for Science. 48013 Bilbao, Spain

[4] Universidad de Buenos Aires. Facultad de Farmacia y Bioquímica. Departamento de Ciencias Biológicas; Junín 956, 1113 Ciudad Autónoma de Buenos Aires, Buenos Aires, Argentina

**Corresponding Authors*

smoya@cicbiomagune.es; david.mecerreyes@ehu.es; miryam.criado@ehu.es

MATERIALS AND METHODS

Materials

Ethyleneglycol diacrylate 90%, diethyleneglycol diacrylate 75%, poly(ethylene glycol) diacrylate average M_n 250 as triethyleneglycol diacrylate, 2,2′-thiodiethanethiol 90%, N-isopropylacrylamide (NIPAM), Darocur 1173®, ketoprofen (KET), and phosphate buffer saline (PBS) were purchased from Sigma Aldrich. Methacrylic acid (MAA), dry dichloromethane 99.8% over molecular sieves, trimethylamine (NEt_3), 1,8-Diazabiciclo[5,4,0]undec-7-ona (DBU), ethyl acetate, and chloroform were purchased from Fisher Scientific. Dulbecco Modified Eagle Medium (DMEM) supplemented with GlutaMAX^{TM}, Penicilin-Streptomicin (5000 U/mL), and trypsin-EDTA (0.24%) phenol red were purchased from Gibco and used as received. Trypan Blue solution, TRITC-phalloidin and dimethyl sulfoxide were purchased from Sigma Aldrich, and Fetal Bovine Serum (FBS) from Life Technologies. Hoechst 33342 was purchased from Invitrogen. CellTiter 96® AQueous One Solution Cell Proliferation Assay (MTS) G3582 was purchased from Promega. All reagents were used as received.

Synthesis of 10,22-dioxo-3,6,9,23,26,29-hexaoxa-13,16,19-trithiahentriacontane-1,31-diyl diacrylate (EG$_3$SA)

The synthesis of the diacrylate thioether monomers was performed via a thiol-Michael addition click reaction following a protocol reported previously.[28] Briefly, in an oven-dried round-bottom flask, 1 equiv. of the desired poly(ethylene glycol) diacrylate was dissolved in dry dichloromethane using 50 mL of solvent for each 3.5 mmol of diacrylate starting materials. To this solution, 2 equiv. of triethylamine and 0.05 equiv. of DBU were added. To the resulting solution, 0.5 equiv. of 2,2′-thiodiethanethiol was added dropwise under continuous stirring and static nitrogen atmosphere, keeping the temperature lower than 25 °C using an ice/water bath. After 4 h, the resulting mixture was put in ethyl acetate, using 250 mL for each 3.5 mmol of starting materials, extracted 3 times with water, using the same amount of ethyl acetate each time, and finally washed with the same amount of brine and, the organic part, dried over anhydrous sodium sulfate. The mixture was filtered, and the solvent was removed under vacuum to afford the pure products. Nuclear magnetic resonance (NMR) spectra were recorded at 25 °C temperature with a 300 MHz Bruker Avance III in $CDCl_3$ (99.5% D)

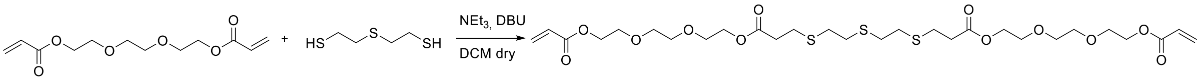

^{1}H NMR (300 MHz, CDCl3) δ 6.48 – 6.36 (m, 2H), 6.14 (ddd, J = 17.3, 10.4, 0.9 Hz, 2H), 5.83 (dt, J = 10.4, 1.8 Hz, 2H), 4.28 (dtd, J = 17.6, 4.6, 1.3 Hz, 8H), 3.80 – 3.56 (m, 16H), 2.88 – 2.69 (m, 12H), 2.64 (t, J = 7.2 Hz, 4H). FT-IR ν_{max}/cm^{-1} 3010 (=C-H), 2953, 2910, 2864, (C-H), 1726 (C=O) and 1445 (C=C), 1109, 856.

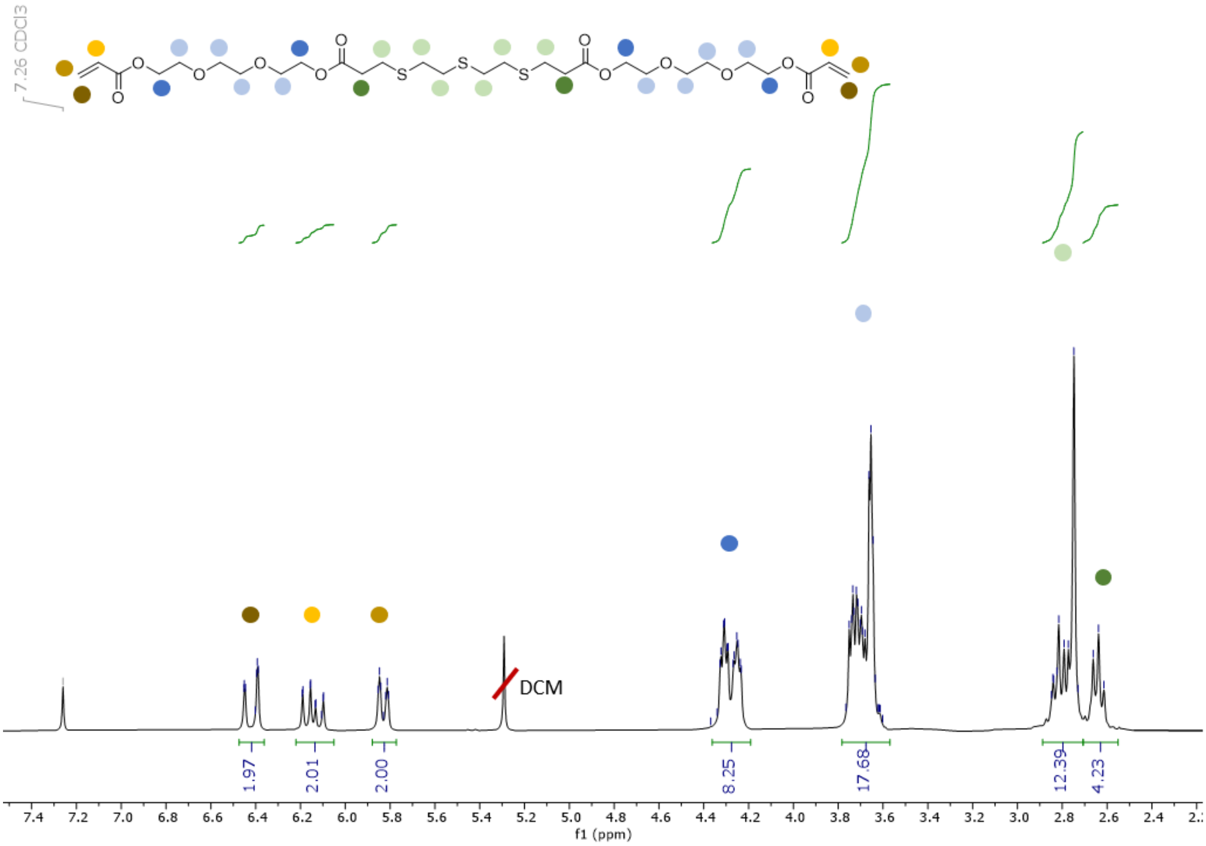

Methods

Hydrogels formation

P[NIPAM$_x$-co-MAA$_y$-co-(EG$_3$SA)$_z$] (x = 40, 70 or 80 %mol; y = 15 or 20 %mol; z = 5, 15 or 40 %mol) hydrogels with different percentages of each monomer were formed in silicon molds of 6 mm diameter and 2 mm height by UV photo-polymerization at 365 nm (80 mW/cm^2). Previously, the monomers were mixed in a vial with 30 %v/v $CHCl_3$ and 10 µL of Darocur 1173®, used as the initiator. Then, the mixture was poured into the silicon mold and irradiated with UV light for 3-5 min depending on the percentage of EG$_3$SA monomer in the mixture.

Fourier Transformed Infrared Spectroscopy (FTIR)

The P[NIPAM$_x$-co-MAA$_y$-co-(EG$_3$SA)$_z$] hydrogels swollen under different conditions, in PBS or 9 mM H_2O_2, at pH 3, 5, 7.4 or 11, and at 25 ºC or 37 ºC were dried before FTIR measurements. Then, FTIR spectra were recorded at each step using an FTIR spectrometer (Bruker INVENIO X).

Swelling tests

P[NIPAM$_x$-co-MAA$_y$-co-(EG$_3$SA)$_z$] hydrogels were washed with PBS for 7 days by replacing the washing solution daily to remove non-reacted monomers. Then, different swelling strategies were tested to prove the responsive effect of each monomer. *i)* The P[NIPAM$_x$-co-MAA$_y$-co-(EG$_3$SA)$_z$] hydrogels were swollen in 1 mL of PBS at pH 5 or 7.4 and at room temperature for 24 h. Subsequently, the hydrogels were swollen under oxidative conditions by immersing them in 9 mM H_2O_2 for 24 h. *ii*) The P[NIPAM$_x$-co-MAA$_y$-co-(EG$_3$SA)$_z$] hydrogels were swollen in 1 mL of PBS at pH 7.4 and room temperature for 24 h. Subsequently, the hydrogels were swollen at 25 ºC or 37 ºC and pH 3, 5, 7.4, or 11, exposed or not to oxidative conditions by immersing them in 9 mM H_2O_2 or PBS for 24 h.

Drug release tests

P[NIPAM$_x$-co-MAA$_y$-co-(EG$_3$SA)$_z$] hydrogels were washed with PBS for 7 days by replacing the washing solution daily to remove non-reacted monomers. First, ketoprofen (KET) was solved in PBS at pH 7.4 (1.5 mg/mL) by sonication for 5 min at 35 ºC and encapsulated into the hydrogels by immersion for 24 h. After that, the supernatant was removed, and KET-loaded hydrogels were washed with PBS to remove the superficial drug and immersed into 1 mL of a fresh PBS solution with and without 9 mM H_2O_2 to start the drug delivery test. At specific times (1 h, 2 h, 4 h, 24 h, 48 h, 72 h, and 168 h), the supernatant was removed and replaced by 1 mL of a fresh PBS solution with and without 9 mM H_2O_2. The quantity of KET in the supernatant was determined by UV-Vis spectrophotometry (Shimadzu UV-2550 spectrometer) by recording the absorbance at 255 nm and comparing it with the KET calibration curve.

Rheological measurements

Photo-rheology was performed in an AR-G2 rheometer (TA instruments) using a UV-light lamp (wavelength = 365 nm, power = 20 mW/cm^2), oscillation stress of 100 Pa, and 0.1 Hz frequency. The samples were placed on a glass parallel plate of 20 mm diameter, letting them stabilize for 60 s to be subsequently irradiated to induce their photopolymerization at 25 ºC.

Frequency sweeps, from 0.01 to 10 Hz at 1% strain, were carried out in an ARES-G2 rheometer (TA instruments) at 37 ºC using a plate-plate configuration with aluminum plates of 8 mm diameter and a gap of 1 mm.

Digital Light Processing (DLP) 4D Printing

The monomeric precursors, NIPAM, MAA, and EG_3SA, were mixed with $CHCl_3$ at 30 %wt and Darocur 1% mmol and poured into the cube basis of the DLP 3D printer (Asiga Max-UV, λ = 365 nm, 20 W/cm^2), and 3D P[$NIPAM_x$-co-MAA_y-co-$(EG_3SA)_z$] hydrogel structures were printed (layer height = 300 µm, exposure time = 30 s). The 4D-printed scaffolds were designed with the Asiga Composer software.

In vitro cell culture tests

Prior to cell seeding, P[$NIPAM_x$-co-MAA_y-co-$(EG_3SA)_z$] hydrogels were placed into a 24-well plate and sterilized under UV light for 1 h. Then, they were washed with 1 mL of PBS under sterile conditions for 7 days to remove non-reacted monomers by replacing the washing PBS solution daily. Subsequently, in the case of drug-loaded hydrogels, they were immersed into 1 mL of a KET solution (0.5 mg/mL in PBS pH 7.4) for 24 h under sterile conditions. Then, the supernatant was removed and the hydrogels were washed with 1 mL of PBS to remove the non-loaded superficial drug. Subsequently, non-loaded and KET-loaded hydrogels were incubated with 1 mL of Dulbecco's modified Eagle's medium (DMEM) enriched with 4500 mg/mL glucose and supplemented with 10% v/v FBS, 2% v/v L-glutamine, 100 units/mL penicillin, and 100 mg/mL streptomycin at 37 °C. The supernatant was removed and replaced by 1 mL of fresh supplemented DMEM after 24 h.

For cell viability assays, mouse embryonic fibroblasts (NIH 3T3) cells or RAW 264.7 murine macrophages (RAW) cells were cultured in DMEM on a 96 well-plate at densities of 1×10^4 cells/well or 2×10^4 cells/well respectively, and incubated at 37 ºC (5% CO_2 and 90% relative humidity) to confluence. After 24 h of incubation, the medium was replaced with the corresponding extracts and incubated at 37 °C in humidified air with 5% CO_2 for 24 h and 48 h. Then, cell viability was estimated by the CellTiter 96® AQueous One Solution Cell Proliferation Assay (MTS) G3582 (Promega, EE. UU.), following manufacturer's instructions. Cells non deposited on hydrogels were considered 100% viable. All experiments were performed in triplicates and the results were expressed as mean ± S.D.

Cell adhesion assays were performed by seeding NIH 3T3 cells following the same culture conditions mentioned for viability studies. After 48 h of incubation at 37 °C and 5% CO_2, hydrogels were washed with PBS twice and fixed with paraformaldehyde 4% for 15 minutes at room temperature. After fixation, hydrogels were washed with PBS and cells were permeabilized using 0.2% PBS Triton X-100 (Sigma) for 5 minutes at room temperature. Another three washing steps were performed using PBS, and cells were stained with TRITC-phalloidin (Sigma) 1/1000 and Hoechst 33342 (5 µg/mL) (Invitrogen) for 15 minutes at room temperature. Finally, hydrogels were washed and kept in PBS for imaging. Confocal scanning laser microscopy images were taken in a Zeiss LSM 900 laser scanning microscope (Carl Zeiss AG, Germany). Images were captured and analysed with the Zeiss Zen acquisition software (Carl Zeiss AG, Germany). A minimum of 10 fields containing several cells were collected.

Nitric oxide (NO) assay for anti-inflammatory properties

For nitric oxide (NO) tests, non-loaded and KET-loaded hydrogels were placed in a 24-well plate. RAW cells were seeded at a density of 2×10^4 cells/well on top of the hydrogels and incubated at 37 °C in humidified air with 5% CO_2. After 24 h incubation, in the case of lipopolysaccharide-stimulated (LPS) RAW cells, 15 µL of LPS (0.5 µg/mL) was added and incubated for another 24 h under the same conditions. Then, NO released by macrophages was determined using the Griess reagent-modified kit (Sigma-Aldrich) according to the manufacturer's instructions. The supernatant (75 µL) was transferred to a 96-well plate, brought in contact with 25 µL of Griess reagent, and incubated for 15 min in the dark. The absorbance was measured in a UV-vis spectrophotometer Cytation Bioteck at a wavelength of 540 nm. Results are shown as mean ± standard deviation of 3 measurements and expressed as a percentage of NO released with respect to the control (LPS-activated cells treated only with medium without hydrogels).

SUPPLEMENTARY FIGURES

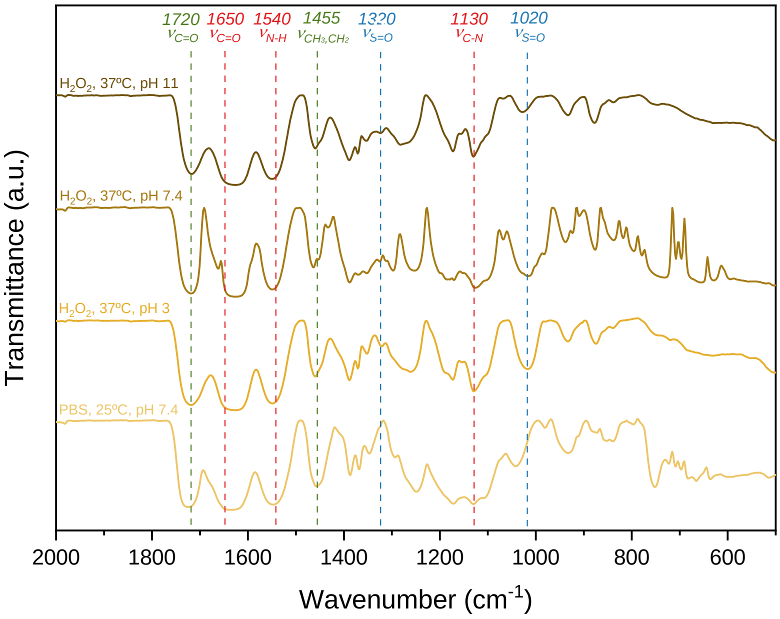

Figure S1. FTIR spectra of P[$NIPAM_{70}$-co-MAA_{15}-co-$(EG_3SA)_{15}$] hydrogels at different temperatures (25 and 37 ºC), pHs (3, 7.4 and 11), in PBS or H_2O_2.

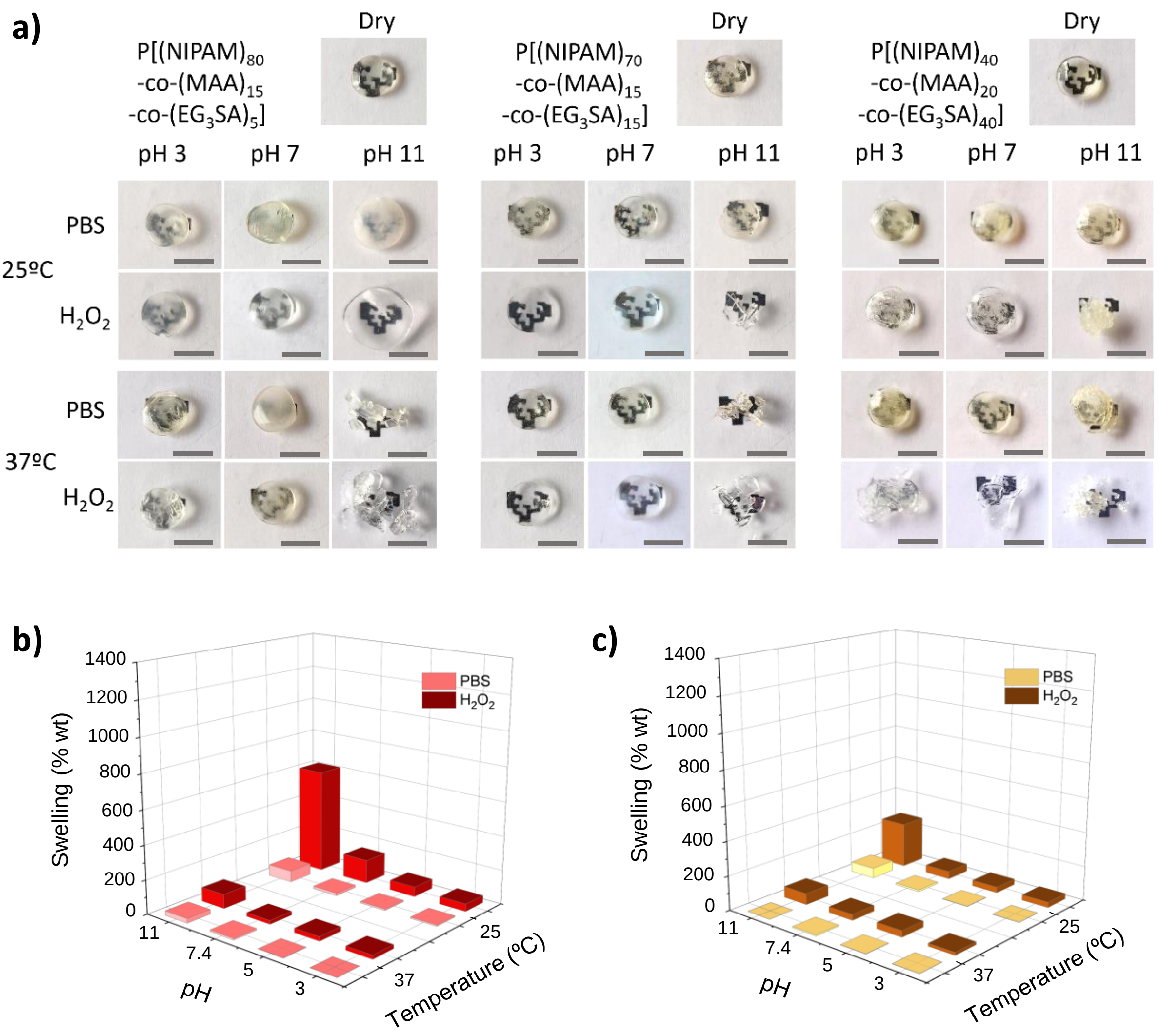

Figure S2. (a) Representative pictures of P[NIPAM$_x$-co-MAA$_y$-co-(EG$_3$SA)$_z$] hydrogels after 24 h at different temperatures, pHs, and under non-oxidant (PBS) or oxidant (H_2O_2) conditions. Scale bars = 5 mm. Swelling comparison of (b) P[NIPAM$_{70}$-co-MAA$_{15}$-co-(EG$_3$SA)$_{15}$] and (c) P[NIPAM$_{40}$-co-MAA$_{20}$-co-(EG$_3$SA)$_{40}$] hydrogels at different temperatures (25 and 37 ºC), pHs (3, 5, 7.4 and 11), in PBS or H_2O_2.

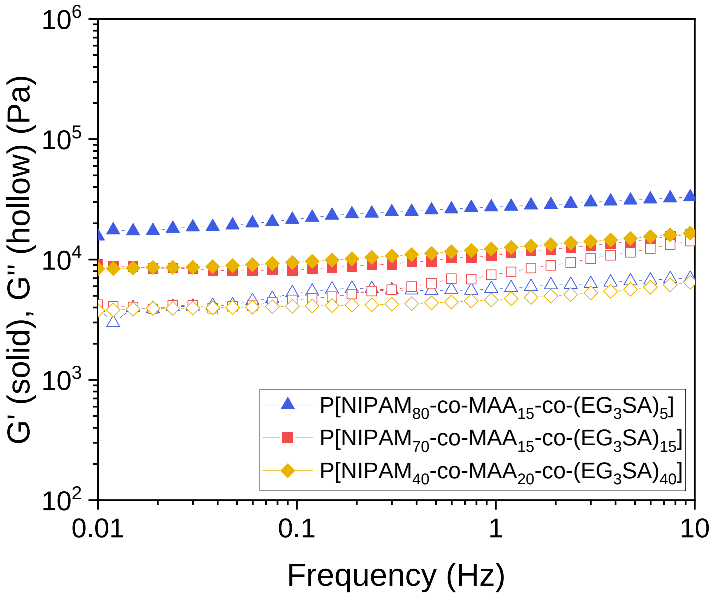

Figure S3. Rheological properties of P[NIPAM$_x$-co-MAA$_y$-co-(EG$_3$SA)$_z$] hydrogels in PBS at pH 7.4 and 25 ºC.

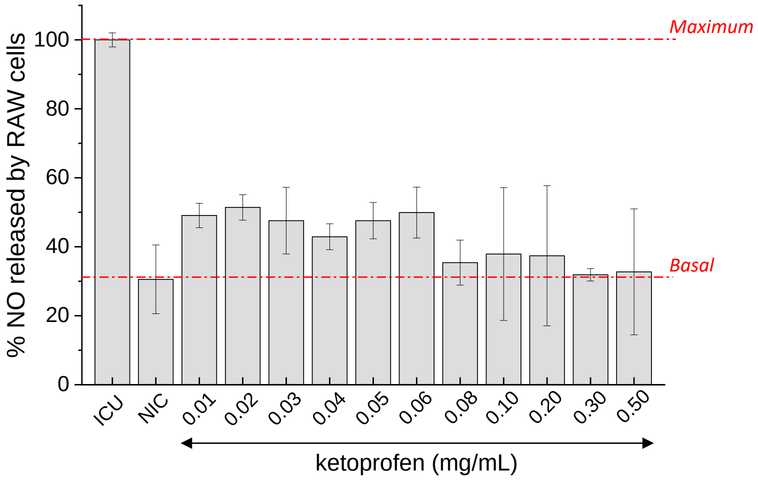

Figure S4. Nitric oxide (NO) released by LPS-RAW cells seeded on the plate (ICU), Non-LPS-RAW cells seeded on the plate (NIC), and LPS-RAW cells seeded on the plate and in the presence of different ketoprofen concentrations. Diagram includes the mean and standard deviation (n = 3).
